# Exploring initiation schools’ impact on HIV and AIDS management in the Vhembe district of South Africa: An ethnography

**DOI:** 10.4102/hsag.v28i0.2105

**Published:** 2023-02-07

**Authors:** Avhatakali A. Ndou-Mammbona

**Affiliations:** 1 Department of Health Studies, Faculty of Humanities, University of South Africa, Pretoria, South Africa

**Keywords:** HIV and AIDS, impact, initiation schools, Leininger’s cultural care modalities, South Africa

## Abstract

**Background:**

This article presents the positive and negative impact traditional initiation schools have on the management of HIV and AIDS in the Vhembe district in South Africa.

**Aim:**

To explore the impact of initiation schools regarding the management of HIV and AIDS.

**Setting:**

This ethnographic study was conducted in rural villages in the Vhembe district.

**Methods:**

Nine purposively sampled key informants from the Vhavenda traditional healers and leaders participated in the study. Data were collected using semi-structured face-to-face interviews guided by an interview and observation guide. Data were analysed using ethnographic content analysis.

**Results:**

The results indicated that the Vhavenda have different traditional initiation schools for boys and girls. For boys, there is *Muḽa* [traditional male circumcision], while *Musevhetho* [first stage of girls’ traditional initiation before puberty], *Vhusha* [girls’ second stage of traditional initiation] and *Domba* [final stage of girls’ traditional initiation] are for girls. Some of the information provided perpetuates engagement in multiple concurrent relationships predisposing them to contract HIV. Boys are encouraged to be strong and to control women when it comes to sexual activities to suit their desire, whether the woman consented or not, while girls are taught to be submissive to their husbands which can fuel the spread of HIV.

**Conclusion:**

As the initiates are attentive to whatever is said during those initiation schools, there is an opportunity for using these initiation schools for proper prevention of HIV and instilling positive behaviours by using Leininger’s cultural care modalities which focus on preservation of beneficial practices and repatterning of practices which fuel the spread of HIV.

**Contribution:**

The study findings will aid in the review and update of the manuals and procedures for HIV and AIDS management.

## Introduction and background

This study investigates the impact of initiation schools on the management of human immunodeficiency virus and acquired immunodeficiency syndrome (HIV and AIDS) among the Vhavenda in the Vhembe district, South Africa. South Africa is one of the sub-Saharan countries where the prevalence of HIV is continuing to rise regardless of the availability of various preventative measures including access to free condom use. In 2000, the prevalence was 12% but it increased to 19% in 2019 (Joint United Nations Programme on HIV/AIDS 2020). However, the HIV prevalence is not the same in the different provinces of South Africa. Limpopo Province, one of South Africa’s provinces, has an HIV prevalence rate of 8.99% (*n* = 151 091). However, the prevalence of HIV in the Vhembe district is 5.4%, which is among the lowest in Limpopo (Vhembe District Health Information System 2019). The reason for this low prevalence is not known. However, in Vhembe, a rural district in South Africa, most of the people attend traditional initiation schools, which may be one of the contributory factors to the low HIV prevalence. A study conducted by Froneman and Kapp (2017) revealed that traditional circumcision for boys plays a role in reducing HIV infections. However, Mutombo, Maina and Jamali (2015) found that male circumcision does not provide complete prevention against HIV infection, and further mentioned that circumcised men can still contract HIV and transmit the virus to their partners. Traditional leaders in the study refers to a person who holds a position in the traditional ruling hierarchy and their role is to preserve customs and traditions (Honig 2019). Traditional healers, on the other hand, are people who use traditional medicine to cure people who are ill or injured and their role is to provide primary care for many people and with training, to be the first stage of diagnosis for some diseases (Chikafu, Mutero & Chimbani 2022). Therefore, the aim of the study was to explore the impact of initiation schools regarding the management of HIV and AIDS.

## Methods

### Design

The researcher followed an ethnographic design for this study. The design was chosen as the researcher wanted to gain insights into the effects of initiation schools on the management of HIV and AIDS among the Vhavenda ethnic groups. In addition, the researcher found that the ethnographic design was more relevant as it allows them to interact with the relevant key informants and the community to understand the initiation practices.

### Study setting

The study was conducted in Vhembe, one of the Limpopo Province’s rural districts, in South Africa. The Vhembe district consists of four local municipalities: Musina, Collins Chabane, Thulamela and Makhado. The Vhavenda people who stay in the Vhembe district are culturally rooted and still engaged in initiation schools. Even if boys undergo medical circumcision, the person will be regarded as a boy until he attended *muḽa* [traditional circumcision] to become a fully-fledged man. Vhembe is the country’s northernmost district and is bordered by Zimbabwe on the northern part and Mozambique on the eastern side (Huffman & Hanisch 2007).

### Population and sampling

The population comprised all the traditional leaders and healers involved in initiation schools. The names of people involved in the initiation schools were provided by the Department of Co-operative Governance, Human Settlements, and Traditional Affairs under the Vhembe House of Traditional Leaders. However, the researcher purposively selected Vhavenda-born traditional leaders and healers aged 35 years and above believed to be knowledgeable and experienced regarding traditional initiation schools in the Vhembe district. Due to the ethnographic nature of the study, the researcher then used snowball sampling by approaching one of the traditional leaders first, and was referred to other individuals who are known as experts in initiation schools to be the key informants in Limpopo. After reaching data saturation at participant number seven, the researcher continued to interview two more participants in order to deduce if there is still new information. In total there were nine key informants involved in the study. The demographic data of the key informants are displayed in Table 1.

**TABLE 1:** Demographic data of the key informants.

Pseudonyms	Age in years	Gender	Traditional practice	Years of practice	Traditional practice observed
Zwoita	82	Male	Traditional healer, initiation school conductor	48	None
Maria	59	Female	Traditional healer, initiation school conductor	27	Virginity testing
Ndiafhi	71	Male	Traditional healer, circumcision school	31	None
Mudzunga	75	Male	Traditional healer, circumcision school	54	None
Muvhuso	54	Male	Traditional healer, circumcision school	42	None
Mutondwa	80	Male	Traditional healer, circumcision school	49	Virginity testing
Muenda	70	Male	Traditional leader, initiation schools (*domba, vhusha* and *musevhetho*)	41	*Musevhetho*
Vele	73	Male	Traditional leader, initiation schools (*domba* and *vhusha*)	33	*Domba*
Ramudzuli	49	Male	Traditional leader, initiation schools (*domba* and *vhusha*)	28	None

### Data collection

Before commencing with the data collection, the researcher developed an interview guide and observation tool based on literature review and the study purpose. The researcher conducted all the interviews, as she is also of Vhavenda origin, and Tshivenda is the mother tongue of the researcher. She has also undergone *musevhetho* [first stage of girls’ traditional initiation before puberty], *vhusha* [girls’ second stage of traditional initiation] and *domba* [final stage of girls’ traditional initiation] and is more conversant with the Vhavenda cultural practices, norms and values. The researcher collected data between June 2019 and December 2019 by using individual semi-structured interviews and an observation tool concurrently following ethnographic design principles. The researcher spent 2–4 days per each practice as per appointment, and permission was given to witness such proceeding in different months. The interviews were conducted face-to-face in the comfort of the particpants’ home in the mornings (from 09:00 to 11:00) on different days when not busy. The following questions were used:

1. What types of initiation schools are practised by the Vhavenda people in the Vhembe district?
2. How are they performed?
3. How can these practices contribute to the management of HIV and AIDS?

Probes and prompts were used following the key informants’ response to elicit more detailed information. All interviews were audio-recorded, and each lasted approximately 45–60 min.

All observations of initiation schools were guided by the following ethnographic observation guide adapted from Roller and Lavrakas (2015):

1. Date.
2. Time of the day.
3. Venue/site.
4. Name of event.
5. People involved.
6. What activities are they engaged in?
7. What are they aiming to accomplish?
8. How exactly are they doing it?
9. What assumptions do they make?
10. What do I observe taking place?
11. What else is happening on the site relevant to the study?
12. How can all the identified activities influence HIV prevention, treatment, and care?

The researcher managed to observe the practices at Musevhethoni, Vhushani and Dombani, which are initiation schools for girls. However, the researcher was not allowed to observe *muḽa* as it is considered taboo for a woman to watch male designated initiation schools. Field notes were also captured during the interviews to describe the environment and non-verbal cues.

### Analysis of data

Data collected through interviews, observation guide and field notes were analysed manually using ethnographic content analysis (Brink, Van der Walt & Van Rensburg 2018; Bryman & Bell 2014) as follows:

Verbatim transcribing of data was done by the researcher after interviews. Translation of interview guide was done by two experts knowledgeable in Tshivenda and English languages. The researcher and an independent coder read all transcripts, field notes and observation tools to code and categorise themes. They then searched for relations across developing themes and grouped similar themes to develop superordinate themes. The researcher developed a master table of themes containing superordinate themes, subthemes and quotes from the transcripts. Then the researcher and the independent coder compared and discussed the table of themes and came up with a finer master table. The process of co-coding independently was done to ensure conformability and reliability (Brink et al. 2018). Three superordinate themes emerged from data analysis, namely, the types of initiation schools conducted by Vhavenda individuals, the practices conducted during initiation schools, and core information provided in the initiation schools.

### Ethical considerations

The Department of Health Studies, REC-012714-039 (NHERC) and the Research Ethics Committee of the the University of South Africa granted the researcher ethical clearance for the study (ethics clearance number HSHDC/902/2019). Permission was also obtained from the Department of Co-operative Governance, Human Settlements and Traditional Affairs under the Vhembe House of Traditional Leaders (Vhavenda Kingship Council). Throughout the study, ethical principles were observed by using pseudonyms to ensure confidentiality and anonymity, obtaining voluntary informed consent from all the key informants, respecting sacred cultural practices, and also adhering to the cultural requirements during data collection such as dressing in a culturally acceptable manner, payment of door and mouth openers (money required when entering the houses of traditional leaders or healers in order to get their permission for the survey), adopting the sitting position which shows respect, such as sitting on a mat made from animal hide, and using Tshivenda when conducting interviews, as the people interviewed were Vhavenda speaking people who were not fluent in English. The researcher only documented information for which permission was given to use as some of the information were regarded as sacred – like male initiation school camps, which is considered taboo for a female researcher to enter.

### Trustworthiness of the study

Guba and Lincoln’s (1994) framework for ensuring trustworthiness was followed to ensure rigour for the study (Polit & Beck 2017). The criteria to ensure trustworthiness included credibility, confirmability, dependability, transferability and authenticity. The researcher has fully described the setting and the study’s key informants to ensure transferability. Interviews were audio-recorded and transcribed verbatim to capture key informants’ voices properly to ensure authenticity and confirmability. The researcher provided feedback to all key informants about the emerging interpretations to obtain the key informants’ responses to ensure credibility. To ensure dependability, the researcher appropriately described the study design, setting, population, sampling, sample, the study’s key informants, data collection and analysis process.

## Findings

The following superordinate themes emerged from the data analysis:

1. The types of initiation schools conducted by Vhavenda individuals.
2. The practices conducted during initiation schools.
3. Core information provided in the initiation schools.

Each superordinate theme has several themes and sub-themes as displayed in Table 2.

**TABLE 2:** Summary of results.

Superordinate themes	Themes	Subthemes
Types of initiation schools conducted by Vhavenda individuals	Initiation schools for boys	*Mu ḽ a*
	Initiation schools for girls	*Musevhetho*
		*Vhusha*
		*Domba*
The practices conducted at initiation schools	The process during *mu ḽ a*	Cutting of the foreskin
		Wound treatment
	The process involved during *musevhetho*	Incisions on the thighs Burning of the thighs
		Singing and dancing
	The process involved during *vhusha*	Virginity testing
		Checking the size of the labia majora
	*Domba* proceedings	Dancing
Core information provided in the initiation schools	Information provided for boys	Being sexually active
		Not listening/being ruled by girls
	Information provided for girls	Being submissive to boys
		Maintenance of virginity until marriage
		Value of staying in marital relationship

Nine key informants participated in this study: eight men and one woman. Six of them were traditional healers and three were traditional leaders. Their ages ranged from 49 to 82 years. One informant was 49 years old, two were between 51 and 60 years, five were between 70 and 80 years, and one was between the age of 81 and 85. The findings indicated that traditional initiation schools for boys and girls are part of the Vhavenda cultural practices that impact the management of HIV and AIDS. The above practices have both negative and positive influences on managing HIV and AIDS.

## Types of initiation schools performed by Vhavenda individuals

This superordinate theme focuses on the types of cultural initiation schools for both boys and girls, which are the following: *muḽa, musevhetho, domba* and *vhusha*.

### Initiation school for boys

For a boy to become a man, he must undergo *muḽa*.

#### Muḽa

Vhavenda boys attend traditional circumcision schools that signify manhood. They are also given rules on how to avoid being infected with HIV and other sexually transmitted infections. According to the Vhavenda culture, a boy child should undergo traditional circumcision (*muḽa*). If not, he will remain a boy until he is traditionally circumcised:

‘Circumcision on the mountain assists in giving rules, not in the hospital where they remain mashuvhuru. That is why there is a lot of bad omens, and HIV is no longer controllable. They have not been given commandments these boys. Boys given rules do not go and spread his seeds all over. He waits to get married before engaging in sexual activities. A married man should first get married and can marry more than one wife. I have married five because I can afford them.’ (Mutondwa, Male, 80 years)

### Initiation school for girls

According to the Vhavenda culture, girls are also expected to attend initiation schools, which are divided into two levels: the primary level is *musevhetho* and the secondary levels are *vhusha* and *domba.*

#### Musevhetho

Girls are expected to undergo a rite of passage to womanhood. At *musevhethoni*, girls are given advice on how to please a man sexually, how to behave and how to be good wives. It is believed that if a girl has undergone *musevhetho,* she will most likely not contract HIV as she was given advice while still young and not yet sexually active:

‘There at musevhethoni, children are given rules about sexuality and how people are supposed to behave when they get married. They are told that a girl should engage only in sex after marriage. And that they should not roam around the street. If this child was following instructions, there would not have been diseases such as HIV/AIDS.’ (Muenda, male, 70 years)

According to the Vhavenda culture, a girl child is expected to attend *musevhetho* before attending *vhusha*, to teach her how she should behave while still young, before she engages in sexual activities.

According to the study it was found that many people think that musevhetho (initiation schools for young girls) is useless. This is due to the fact that some people do not know what is happening there:

‘That’s where young girls get rules on how people should behave. The good thing is that they are given instructions while they are still young. We tell them not to engage in sexual activities while they are still young and that the diseases are so powerful. HIV can be reduced if children follow the instructions while they are still young. This tradition of us is very rich. There at musevhethoni we can see if the child is still a virgin.’ (Maria, Female, 59 years)

After attending *musevhetho*, girls will proceed to the secondary level of traditional initiation schools, namely *vhusha* and *domba.*

#### Vhusha

Here girls are encouraged not to engage in sexual intercourse before marriage to avoid contracting sexually transmitted infections such as HIV. If found to be already sexually active, they are encouraged to use condoms to avoid re-infection and to curb the spread of HIV to others:

‘When older girls go to vhushani that is where they are taught how to respect their husbands and to remain faithful in the marriage and that they should not engage in sexual intercourse with men as they will get infected with HIV. If they are already sexually active, they are advised to use condoms to avoid being infected with HIV.’ (Maria, Female, 59 years)

#### Domba

For an older girl to be fully initiated, she must attend both initiation schools. Older girls are given advice at *dombani* on how to behave while married. They are also instructed on how to satisfy a man sexually and told to love their husbands no matter what. Women are expected to be always faithful to their husbands. They are advised to only engage in sexual intercourse after marriage:

‘At dombani, girls are given instructions to love their husbands and that they should be faithful. Here at domba, there is a song called mabidigama. Here people are being given instructions about sexual intercourse and that it is done only by married people.’ (Mutondwa, Male, 80 years)

## The practices conducted at initiation schools

### The process during *muḽa*

Even though the process of *muḽa* is sacred and cannot be witnessed by a girl, one of the initiators of *muḽa* explained the process to the researcher – read the following excerpt:

‘You want to know how I circumcise. This is what I do. The razor is cleaned with a methylated spirit as to use one razor per person is impossible because the initiates are more than hundred. However, they are protected. We just use the razor per person because of the interference by the Department of Health. But the truth is that the way we do this, even if we are using one knife, nothing will happen. I have been handling circumcision school since I was only 16, but no one ever gets a sexually transmitted infection from the circumcision school.’ (Zwoita, Male, 82 years)

#### Cutting of the skin

In the Vhavenda culture, during *muḽa*, only a knife consecrated by the initiation traditional healer is used for all the initiates:

‘We cut their foreskin with a knife which has also been consecrated by the same traditional healer who has consecrated the circumcision school. All the foreskins are mixed with a medicine known by the traditional healer and are burned to make a treatment for circumcision wounds. Just by applying the mixture, (muuluso), the wound becomes immediately healed. The wound never becomes septic.’ (Ndiafhi, Male, 71 years)

While one of the key informants mentioned using one knife to cut the foreskin of all the initiates, another key informant mentioned the use of one razor per initiate:

‘I use one knife to cut all initiates’ foreskin, and they will never get infected with infections like HIV. I have been in this initiation thing for so many years and I have never seen any of them contracting HIV, never in my life. I am also thinking of engaging the health department of test all my initiate before and after under the consent of the parents.’ (Mudzunga, Male, 75 years)

‘I am careful when cutting the foreskins, I use one razor to one child. I don’t buy them, because they are many, all the parents of the initiates bring the razors to me, and I consecrate them before performing traditional circumcision. Some boys are born being infected with HIV, some come to muḽani already being sexually active, so you will never know who is infected with HIV or not. To be safe, I adhere to infection prevention and control practices.’ (Ramudzuli, Male, 49 years)

#### Wound treatment

The issue of circumcision school does not only end in the bush where *muḽa* is hosted, but it also extends to home after the initiates are released. This fact was supported by the following excerpt of a key informant:

‘Only the muuluso smeared in the incised part at circumcision school is enough to heal the wound. When initiates come from circumcision, nobody can be in the hut/room where they are, and the mother is not allowed to see the private part. No life has been lost since I started initiating (protecting) the circumcision schools. I first officially inform the ancestors so that they open the way, and everything goes on well.’ (Zwoita, Male, 82 years)

### The processes involved during *musevhetho*

At *musevhethoni,* incisions are made on the thigh of all girls who are initiated using the same knife, as it is believed that the traditional healer also purifies the knife of choice. It is believed that no one has ever been infected with HIV since the inception of traditional schools like *musevhethoni* – according to the knowledge of traditional healers and traditional leaders:

‘Since birth I have never seen a child who is said to have been infected by HIV during musevhetho. The knife is washed using the traditional medicine before being used for another child. In fact, it is not every knife, that one is first sanctified by the traditional healer so that there will be no child who contract the disease or become septic.’ (Muenda, male, 70 years)

#### Incisions done on the thighs

Girls are incised on the thighs to symbolise that they have undergone *musevhetho*. The process itself is painful and is done in the river using one razor for all the girls:

‘Girls are cut like “11” [*eleven*] on the middle or sides of thighs and be burned on top of “11” [*eleven*] for a circle-shaped figure. We use one knife in the river sanctified by the chief’s traditional healer, that is why children will never get infected with HIV. The river water becomes red with blood.’ (Muenda, male, 70 years)

#### Burning of the thighs

On top of the ‘11’, the thighs are then burned using burning wood to make a round shape, concluding the traditional ritual. The following participant’s excerpt attests to the above:

‘I use one consecrated burning wood to burn their thighs. It should be like that; it symbolises that the girl is fully initiated regarding musevhetho. I have never infected any girl with HIV.’ (Maria, Female, 59 years)

After the procedure, there will be singing of songs and dancing symbolising that the deed has been done.

#### Singing and dancing

Songs will be used to give rules to all the initiates and the initiates will dance while singing the rules (words) – it will tell them that it is not right to engage in sexual intercourse before marriage, and they are also advised to remain virgins until they are married:

‘In every dance and song there are rules attached to guide the young girls in the ways of life, how they should behave, how to abstain from sex, teaching them that sex is good when married only, and they should not be sexually active before marriage as they will contract illnesses like HIV/AIDS and die still young.’ (Zwoita, Male, 82 years)

### The processes involved during *vhusha*

At the traditional initiation schools, from *muḽani, vhushani* to *dombani,* the girls are subjected to inspection to see whether they are still virgins.

#### Virginity testing

The findings reveal that girls at Vhushani are being tested to see if they are still virgins and they are rewarded if they are still virgins. Those who are already sexually active are advised to use condoms to decrease the spread of HIV and to minimise infections:

‘Girls entered the room one by one and two elderly women are assessing them as to whether they have yet engaged in sexual activities. They check if the hymen is still intact. If found still a virgin, they are given a calabash which is not broken to show that they are a virgin; if no longer a virgin, they are given a broken calabash. The whole process is done inside the house where each girl’s legs and private parts are covered with a clean cloth.’ (Maria, Female, 59 years)

‘The other thing that can help is to send all girl children for virginity testing by the community elders. We will find fewer teenage pregnancies and less people living with HIV/AIDS. If more virginity tests are done, girls will make sure that they remain virgins until they marry their husbands. The above test will also make the girls faithful to not disappoint themselves and their parents. There should be a way to award those who remain virgins to encourage other girls to follow in their footsteps.’ (Muvhuso, Male, 54 years)

Not only the status of the hymen is inspected, but they also check if the girl has pulled her *eleven*.

#### Checking of *labia majora*

A longer pulled *labia majora* is believed to ensure that the girl will sexually appease her husband, but the elders are also on the lookout for sores around the *eleven*, symbolising if the girl has sexually transmitted infections, including HIV before marriage.

### Domba proceeding

The process done at *dombani* is sacred, but the researcher was fortunate enough to witness the *domba* proceedings after paying *mvulamulomo* [mouth opener] of R50.

#### Dancing at *dombani*

The older girls at *dombani* are taught how to please a man sexually and how to take care of their husbands, which is also shown while dancing wearing *mashe ḓ o* [Venda traditional attire which covers only the front part of the private parts and the anal cleavage and leaves the side of the thighs and buttocks exposed.]. The following participant’s transcript detailed how the *domba* proceedings are carried out:

‘They put on *masheḓo* and they dance holding each other’s arms making a chain.’ (Zwoita, Male, 82 years)

‘Yes, they are in contact with men while wearing masheḓo. Remember that the main purpose is for them to get knowledge about sexual activities and how to care for a man.’ (Zwoita, Male, 82 years)

## Core information provided in the initiation schools

According to the Vhavenda tradition, a real man respects his wife or wives and will not cheat as he is allowed to marry more than one wife, which means that he will always be satisfied sexually and will have no time for cheating. This tradition should also prevent a husband from contracting and spreading HIV and AIDS to his family:

‘Circumcision on the mountain assists in giving rules, not in the hospital where they remain mashuvhuru. That is why there is a lot of bad omens, and HIV is no longer controllable. Boys given rules do not go and spread his seeds all over. He waits to get married before engaging in sexual activities. A man should first get married and can marry more than one woman. I have married five because I can afford them.’ (Mutondwa, Male, 80 years)

### Information provided for boys

Boys at the initiation schools are advised on the importance of remaining faithful to their wives to reduce the spread of HIV and AIDS:

‘Traditional circumcision is helping a lot because that is where boys are being given traditional values of life. They are taught the appropriate behaviour. They are taught that a good man will respect his wife/wives. The right men will never cheat but are allowed to marry more than one wife to avoid getting infections such as HIV due to unfaithfulness or cheating. If he marries more than one woman, there is no need to cheat as he will always be busy and satisfied sexually at home.’ (Vele, Male, 73 years)

#### Being sexually active

At Vhavenda initiation schools, boys are taught to abstain or protect themselves when engaging in sexual intercourse to avoid being infected with HIV and AIDS. If not, they are encouraged to marry more than one woman:

‘Boys are told that it is ok to marry more than one woman, so that they cannot starve themselves sexually. They are made to believe that there is no way they can be sexually satisfied by one woman. To avoid cheating, it is deemed fit for a man to marry more than one wife, so that he can remain faithful and not bring HIV home. Boys are taught at the circumcision schools that they should use condoms when engaging in sexual activities to avoid contracting HIV. In fact, we teach them that they should not engage in sexual activities until they get married. All these are done for self-protection from HIV/AIDS. We teach them true morals, and when they come out, they are mature.’ (Zwoita, Male, 82 years)

Men always have the upper hand when compared to women, as they are regarded as powerful and strong, whereas women are regarded as followers of men.

#### Not listening to or being ruled by girls

The study revealed that, as culture permits, men may marry more than one woman without first consulting their wife or wives. This is to show supremacy towards the women. A wife will never ask her husband to use condoms to protect herself from being infected with HIV but will engage in sexual intercourse with her husband even if he is not faithful and risk her life:

‘You know that during traditional circumcision boys are taught to be men and not to be ruled by the women they marry. If the husband wants his women, he must be given what is his, I mean sex; no negotiation, unless the woman is having her period [*menstruating*] or sick.’ (Muvhuso, Male, 54 years)

### Information provided for girls

While young girls at *musevhethoni* are given advice on acceptable behaviour, older girls are taught how to respect, take care, and remain faithful to their husbands so that they would not be infected with HIV and AIDS. The following key informants attest to the above:

‘A lot of people think that musevhetho (initiation schools for young girls) is useless. That’s where young girls get rules on how people should behave. The good thing is that they are given instructions while they are still young. We tell them not to engage in sexual activities while they are still young and that these diseases are so powerful. This tradition of us is very rich. There at musevhethoni we can see if the child is still a virgin.’ (Mutondwa, Male, 80 years)

‘When older girls go to vhushani that is where they are taught how to respect their husbands and to remain faithful in the marriage and that they should not engage in sexual intercourse with men as they will get infected with HIV. If they are already sexually active, they are advised to use condoms to avoid being infected with HIV.’ (Maria, Female, 59 years)

#### Being submissive to boys

Girls are taught to always be submissive to their husbands – even if their husbands are having extramarital affairs known to them, they should continue sleeping with them without negotiating about wearing condoms and risk their lives by contracting HIV and other sexually transmitted infections. The study also revealed that women are more prone to contract HIV from their husbands due to extramarital relationships:

‘In Tshivenda culture the man as the head of the family will always be superior to the woman. A woman cannot deny her husband sex, even if the husband is having concubines. You may find that the husband is cheating because the woman is not submissive to him. Even the bible say women must submit to their husband and the husband must love his wives.’ (Ndiafhi, Male, 71 years)

#### Maintenance of virginity until marriage

Rewards or presents are offered during virginity testing to girls who are still virgins to encourage them to remain virgins until marriage:

‘During domba and vhusha they are given instructions on behaviour. A female should not roam around as if she is selling tomatoes but must stay at home until people come and pay lobola. At vhushani, mature adolescents are given rules to love their husbands and to be faithful. We teach them about sexually transmitted infections, including HIV, and that if they engage in sex before marriage, they can contract HIV and have unplanned babies. Girls are inspected and those who are still virgins are given rewards such as a computer to use when they continue with their studies. It makes others to admire them for getting computers. This is to prevent them from engaging in unprotected sex and contracting HIV.’ (Ramudzuli, Male, 49 years)

According to the Vhavenda culture, women are expected to stay married even if their husbands are not faithful and the wife is predisposed to sexually transmitted infections, including HIV and AIDS.

#### Value of staying in marital relationships

According to Vhavenda cultural beliefs, there is a saying that a woman looks beautiful while staying in her husband’s house, whereas another idiom says that ‘the woman’s grave is found at her husband’s kraal’. For a woman to gain dignity, she should stay with her husband even if the husband is having extramarital affairs – and she is aware of his unfaithfulness. The following key informant’s quotation was found to be appropriate:

‘A women must not leave her husband because of concubines. That woman will lose her worth as she will be referred as a failure. If she engages in sexual intercourse with other men, she will contract HIV in the process. It is better for her to stay at her husband’s house.’ (Mudzunga, Male, 75 years)

## Discussion

As the aim of the study was to explore the impact of initiation schools regarding the management of HIV and AIDS, the study’s findings have highlighted some of the positive and negative impacts that Vhavenda traditional initiation schools have on the management of HIV and AIDS in one of the rural districts in South Africa. The study’s findings identified several risky cultural practices and beliefs among the Vhavenda people in the Vhembe district, which impact the management of HIV and AIDS. The study also identified how traditional circumcision can positively impact the transmission of HIV and AIDS, as men who are circumcised are less prone to contract HIV than men who are not circumcised (Prodger & Kaul 2017).

## Types of initiation schools conducted by Vhavenda individuals

The study indicated that traditional circumcision is done in a non-hygienic environment and some traditional leaders use only one knife for all initiates as it is believed to be sanctified by the ancestors. This has the potential to transmit diseases, such as HIV and AIDS. According to the above study, Maffioli (2017) found that traditional circumcision is contributing negatively to the management of HIV and AIDS as it is performed in an unhygienic and non-clinical environment. Contrary to the above is the findings in the study by Banda and Kunkeyani (2015), that in Malawi, there is a change on how they conduct the traditional initiation ceremonies. It is now done in a safer environment and instead of using one razor blade for circumcising many boys, these days one blade is used for one boy and then thrown away for fear of the transmission of HIV.

In the study it was also revealed that girls also undergo musevhetho as the primary level. At musevhethoni, incisions are made on the thighs of girls using a razor and burns made on their thighs using burning wood. The above practices can predispose them to contract HIV, if some of the girls are already HIV positive. Sharing the same narrative is the study conducted by Tshifhumulo (2022) that incision done to children at *musevhethoni* can predispose them to contract HIV and AIDS, as they often use one knife to all children. After musevhetho, the older girls proceed to the secondary level, which is *vhusha* and *domba* where they are given rules and guidance on how to abstain until they become married and how to behave when married. Attesting to the above is the study conducted by Mudau et al. (2018), that vhusha and domba help in grounding older girls to abstain from sexual intercourse before marriage.

## The practices conducted during initiation schools

The study revealed that traditional circumcision can reduce HIV infections as men who are circumcised are less prone to be infected with HIV. Contrary to the above, a study conducted by Maffioli (2017) found that 59% of men who have undergone traditional circumcision realised that a part of the foreskin was not removed; therefore, only those who underwent male medical circumcision are fully circumcised and are less prone to sexually transmitted infections, including HIV and AIDS. The study also indicated that men who have undergone traditional circumcision believe that they are immune to contracting HIV and AIDS even when they engage in unprotected sex. Testimony to the above statement is that circumcised men in Malawi have a higher HIV prevalence rate (12%) than the uncircumcised men (10%), because after traditional circumcision they engaged in sex with multiple sexual partners without using condoms as they believed that they are not prone to HIV and other sexually transmitted infections (Mutombo, Maina & Jamali 2015).

The study also found that at *musevhethoni, vhushani* and *dombani,* girls undergo conventional virginity testing, which has rewards for those who are still virgins. Those who are already sexually active are advised to use condoms to prevent them from contracting HIV and AIDS. In Zimbabwe, virginity testing is performed to curb the further spread of HIV and AIDS (Thobejane & Mdhluli 2015). The testing is carried out to encourage total abstinence from sexual intercourse by unmarried girls; the practice is being revived to prevent HIV infection and detect incest and abuse, and to re-instil and promote lost cultural values (Thobejane & Mdhluli 2015).

The men believe that they are strong and more superior than women. Men also believe that they can do as they please because women should submit to them even if they know that they are not faithful and probably infected with HIV. Sharing the above sentiment is Gazimbi (2019), that traditional circumcision includes rites of passage, blood sacrifices, cultural markings, enhancement of masculine fearlessness and fecundity, preparation for marriage and adult sexuality, and the hardening of boys for warfare.

## Core information provided in the initation schools

Boys are given rules on how to behave and not to engage in sexual intercourse while not married and to remain faithful to their wives. As attested by Douglas and Hangoro (2018), the initiates stay in the initiation school for about 3–4 weeks to observe all the cultural rites, including learning about sexual, individual, and community values. They are also taught about the dangers of engaging in promiscuous behaviour as one can contract HIV and AIDS in the process. Testimony to that is the study conducted by Rathebe (2018), where he reported that boys spend time in the mountains to be taught about their culture, respect and most importantly, about diseases. If they have extramarital sexual relations without protection, they may be infected with HIV and AIDS.

Another cultural practice having a positive influence on the management of HIV and AIDS is when girls attend traditional initiation schools where they receive rules and guidance concerning ways of life and are encouraged to remain celibate until they become married. Girls are given advice which have a bearing on their future. Attesting to the above statement is the study conducted by Mdhluli et al. (2021), that initiation schools for boys and girls are viewed as educational institutions where initiates are educated about courtship, marriage negotiations, their social duty and how to conduct themselves as men, as well as being circumcised.

Girls are expected to wait until marriage before engaging in sexual intercourse, and to avoid being infected with HIV and other sexually transmitted infections which are detrimental to their health. Rathebe (2018) believes that young girls who attend these schools should be taught the values, principles, hardships, respect and accountability of their cultural tradition.

## Limitation of the study

The limitation of the study is that it was only conducted in the Vhembe district, whereas Limpopo has four other districts, namely Mopani, Waterberg, Sekhukhune, and Capricorn. The key informants in the study were voluntary participants and targeted traditional leaders and healers of the community who met the inclusion criteria. These criteria excluded Vhavenda community leaders and elders who are involved in initiation schools but were not willing to participate and those involved in traditional initiation schools but did not stay in the Vhembe district during the data collection. However, the author has thoroughly explained the measures followed to ensure the study’s trustworthiness. This is a limitation as the Vhembe district is composed of Vhavenda people and people from other areas and countries who also relate to the Vhavenda people in the district. The researcher used interviews and only three observations to collect data, which is a limitation as ethnographic studies depend to a great extent on observations. However, the observation of different cultural activities was not permitted as some activities carried out are regarded as sacred. Another reason was that someone should first undergo these rituals to observe certain rituals. As the researcher was a woman, she was not permitted to witness rituals such as traditional male circumcision. Some of the traditional practices such as *vhusha* are carried out annually, so the period fell outside the researcher’s data collection period. The observation done was for virginity testing, *musevhethoni* proceedings and *domba* proceedings which the researcher was given permission to observe after paying *mvulamulomo* (mouth opener) of R50 for each observation.

## Conclusion

The study explored Vhavenda traditional schools, which may influence the management of HIV and AIDS. The study’s results shed light on the enhancements recommended for education and training, nursing practice and further research. The study also contributed to the body of knowledge about integrating cultural practices into HIV and AIDS management. The findings will also guide traditional healers and leaders in the proper management of HIV and AIDS. Even though there are some unsafe Vhavenda cultural practices prompting the spread of HIV and AIDS in the Vhembe district, which need to be repatterned following Leininger’s modalities of care, the results will aid in re-modelling risky cultural behaviours of the Vhavenda and have the following desired outputs: safe cultural practices, the reduction of new HIV infections, and the reduction of HIV and AIDS-related deaths. The findings can be used in other African countries which have similar traditional initiation schools practices.

## References

[CIT0001] Banda, F. & Kunkeyani, T.E., 2015, ‘Renegotiating cultural practices as a result of HIV in the eastern region of Malawi’, *Culture, Health & Sexuality* 17(1), 34–47. 10.1080/13691058.2014.944569 25138154

[CIT0002] Brink, H., Van der Walt, C. & Van Rensburg, G., 2018, *Fundamentals of research methodology for health care professionals*, 4th edn., Juta, Cape Town.

[CIT0003] Bryman, A. & Bell, E., 2014, *Research methodology: Business and management contexts*, Oxford University Press, Cape Town.

[CIT0004] Chikafu, H., Mutero, I.T. & Chimbari, M.J., 2022, ‘“If i were to suffer a stroke right now, the first place that i should be taken to is the traditional healer”: Community beliefs and health-seeking practices for noncommunicable diseases in Rural KwaZulu-Natal, South Africa’, *The Qualitative Report* 27(1), 243–256. 10.46743/2160-3715/2022.5229

[CIT0005] Douglas, M. & Hongoro, C., 2018, ‘The consideration of socioeconomic determinants in prevention of traditional male circumcision deaths and complications’, *American Journal of Men’s Health* 12(3), 597–607. 10.1177/1557988316638157 PMC5987959 26993997

[CIT0006] Froneman, S. & Kapp, P.A., 2017, ‘An exploration of the knowledge, attitudes and beliefs of Xhosa men concerning traditional circumcision’, *African Journal of Primary Health Care & Family Medicine* 9(1), 1–8. 10.4102/phcfm.v9i1.1454 PMC5645564 29041800

[CIT0007] Gazimbi, M., 2019, ‘The association between male circumcision and HIV infection in sub-Saharan Africa: A systematic review of the literature’, *International Archives of Public Health and Community Medicine* 3(1), 14–31. 10.23937/2643-4512/1710022

[CIT0008] Guba, E.G. & Lincoln, Y.S., 1994, ‘Competing paradigms in qualitative research’, in H. Giroux & R. Stake (Eds.), *Handbook of qualitative research*, vol. 2, no. 163–194, p. 105, Sage, London.

[CIT0009] Honig, L., 2019, ‘Traditional leaders and development in Africa’, in W.R. Thompson & E.M. Kuhonta, *Oxford research encyclopedia of politics*, pp. 163–194, Oxford University Press, Cape Town.

[CIT0010] Huffman, T.N. & Hanisch, O.M., 2007, ‘Settlement hierarchies in the Northern Transvaal: Zimbabwe ruins and Venda history’, *Journal of African Studies* 46(1), 79–116. 10.1080/00020188708707665

[CIT0011] Joint United Nations Programme on HIV/AIDS, 2020, *Global AIDS*, viewed 20 August 2022, from http://www.uniaids.org/sites/default/media. 12349391

[CIT0012] Maffioli, E.M., 2017, ‘Is traditional male circumcision effective as an HIV prevention strategy? Evidence from Lesotho’, *PLoS One* 12(5), e0177076. 10.1371/journal.pone.0177076 28498835 PMC5428932

[CIT0013] Mdhluli, T.D., Matshidze, P.E., Kugara, S.L., Vuma, L. & Mawere, J., 2021, ‘An investigation into the commercialisation of initiation schools: A case of Eastern Cape, South Africa’, *HTS Teologiese Studies/Theological Studies* 77(2), 1–8. 10.4102/hts.v77i2.6157

[CIT0014] Mudau, N., Matshidze, P., Netshandama, V. & Masoga, A., 2018, ‘Reflections on practices of U laya nwana: Towards an Afro-sensed approach’, *African Renaissance* 15(1), 181–195. 10.31920/ROPO_15_1_18

[CIT0015] Mutombo, N., Maina, B. & Jamali, M., 2015, ‘Male circumcision and HIV infection among sexually active men in Malawi’, *BMC Public Health* 15(1), 1–9. 10.1186/s12889-015-2384-z 26463045 PMC4605099

[CIT0016] Polit, D.F. & Beck, C.T., 2017, *Nursing research: Generating and assessing evidence for nursing practice*, Williams & Wilkins, Philadelphia, PA.

[CIT0017] Prodger, J.L. & Kaul, R., 2017, ‘The biology of how circumcision reduces HIV susceptibility: Broader implications for the prevention field’, *AIDS Research and Therapy* 14(1), 1–5. 10.1186/s12981-017-0167-6 28893286 PMC5594533

[CIT0018] Rathebe, P.C., 2018, ‘The role of environmental health in the Basotho male initiation schools: Neglected or restricted?’, *BMC Public Health* 18(1), 1–8. 10.1186/s12889-018-5936-1 PMC6085624 30092784

[CIT0019] Roller, M.R. & Lavrakas, P.J., 2015, *Applied qualitative research design: A total quality framework approach*, Guilford Publications, New York, NY.

[CIT0020] Thobejane, T. & Mdhluli, T., 2015, ‘Probing the efficacy of virginity testing on the fight against HIV/AIDS: The case of the Kwa-Zulu Natal, South Africa’, *OIDA International Journal of Sustainable Development* 8(7), 11–20.

[CIT0021] Tshifhumulo, R., 2022, ‘Depicting the Vhavenda Women Initiation schools and their cultural practices in Limpopo Province’, in R. Tshifhumulo & T.J. Makhanikhe (eds.), *Handbook of research on protecting and managing global indigenous knowledge systems*, pp. 341–364, IGI Global, IGI Global.com, Hershey, Pennsylvania.

[CIT0022] Vhembe District Health Information System, 2018, *Annual report*, Government Printers, Pretoria.

